# Progress in Surgery

**Published:** 1899-11-04

**Authors:** 


### PROGRESS IN SURGERY.

### HERNIA.

**The Radical Cure of Inguinal Hernia.** — Dr. J. O'Conor,[1] writing on this subject, alludes to 150 operations of the kind he has performed. Six were by Barker's method, 129 by Halsted's, and the last 15 by a method of his own. Criticising Halstead's operation this writer says that as far as slight mortality and permanency of result goes there is little to find fault with, but against the operation is the fact that in 80 per cent. of the writer's cases orchitis followed it, and in 20 cases atrophy of the testicle supervened. O'Conor asserts that Italian surgeons report that a similar effect on the testicle frequently follows Bassini's operation. This is the reason O'Conor has introduced a new method. In this the chief object aimed at is the obliteration of the funnel-shaped peritoneal process, and the increase of the resistance offered by the transversalis fascia to the descent of hernia at the internal ring. The operation is as follows: A three-inch incision is made, beginning half an-inch internal to the anterior superior iliac spine and ending an inch above Poupart's ligament. The muscles are opened by separation of their fibres, as in McBarney's appendicectomy. The funnel-shaped process of the peritoneum is then sought for, separated from the cord, incised, and its contents reduced, ligatured below the funnel, and divided on the proximal side of the ligature. The distal portion of the sac is allowed to drop back into the inguinal canal and is left there. It is not considered expedient to divide muscles to remove it. The cut edges of the peritoneum are united by a continuous catgut suture. Next the edges of the opening in the transversalis fascia, which is always found to be very large, are drawn into the field of operation; from four to six silkworm gut sutures are then passed through the external oblique (internal?) and transversalis muscles, and brought out in inverse order on the opposite side, room being left for the cord. The wounds in the external oblique and skin are united by separate continuous catgut sutures. It is remarked that the natural anatomical support is not weakened in this operation by division of muscle fibres, and that all superfluous surgery is avoided.

Brenner[2] suggests a slight modification of technique in Bassini's operation, which he has used, and which has yielded very good results. The modification is, that in opening up the canal, care is taken to separate the cremaster muscular fibres from those of the internal oblique, and both from the aponeurosis of the external oblique. After raising the cord these muscular fibres are united behind it. It is maintained that the union resulting between muscular and muscular tissues, in this modification, is much firmer than where the internal oblique and cremaster are united to the fibres of Poupart's ligament.

The author believes in using a drainage tube for 24 hours. He has operated upon 348 herniæ. The majority of patients were dismissed in 14 days without a truss. Examination of 169 of these one year and more after operation showed relapse in 10 cases, *i.e.*, 5·9 per cent. If there is weakness on the other side, or incipient hernia in young adults, he recommends operation on that side also. Dr. Emory Lanphear[3] describes a new operation for radical cure. A large flap is turned back exposing the hernial sac and inguinal canal in their entirety. The sac is opened, its contents reduced. An artificial tunica vaginalis is made from the sac, and the testicle and cord displaced into it and enclosed with sutures; the whole is then pushed into the abdominal cavity and anchored by sutures. The peritoneum, and each muscular layer of the canal are sutured, and thus the canal completely obliterated. Objections to the operation are the possibility of atrophy, or of inflammation of the testicle supervening. Dr. A. H. Ferguson[3] considers his operation for hernia the ideal one, because it places all structures in the same relation with one another as in normal persons. A semilunar incision was used, the flap being turned down. If relapse occurred there would thus be good sound skin for a truss. The external ring was split and the sac emptied and removed. A new internal ring was then made, using the transversalis fascia. The cord was left alone. The internal oblique was sutured to Poupart's ligament, *i.e.*, putting it where it belonged. Dr. Lee[3] says, that in his opinion, most, if not all, surgeons met with more or less suppuration in hernia operations. The skin, he points out, is difficult to disinfect. Infection was early or late—the former coming on in two or three days, and being due to infection at the time of operation; the latter after two or three weeks. He does not think that tension of sutures has anything to do with this. Two to two and a half weeks was the limit in which a suture was of any value whatsoever.

**The Radical Cure of Umbilical Hernia.** — Howard Marsh[4] has written on this. He advocates a modification of the usual methods, applicable whether the hernia is strangled or not. Instead of opening the sac by a vertical incision through it and the overlying skin, he makes a curved incision through the skin on each side, starting in the middle line above and including the skin over the centre, and only leaving enough at the sides to allow of closure of the wound subsequently without tension. Next, with a sweep of the finger and a few touches of the knife, the whole thickness of the subcutaneous tissue is separated on either side from the outer surface of the sac and its neck, which are thus clearly defined. The sac is now opened in the middle line freely, and the omentum unfolded—any part may be divided between forceps if not near the neck of the sac—and the intestine exposed. Adhesions of the intestine to the omentum or sac are then separated. It is safer and quicker, if the adhesions of the intestine are dense, to cut through them at a little distance from the bowel, and return the latter with portions of them upon it. To save time in dealing with the omentum its neck is defined where it emerges from the abdomen, and is freed from adhesions there. This stem is then tied in strands and returned. The sac, containing perhaps a large mass of adherent omentum, is then removed flush with the ring, and the peritoneal edges sutured. Then the ring is separately sutured, and the skin. The method of dealing with the omentum at its stem, instead of detaching all the adhesions peripherally, probably curtails the length of the operation by a half.

**Partial Enterocele.**—Mr. W. H. Battle[5] relates a case of this. The patient, a woman of 29, first had abdominal pain on April 10th, coming at intervals. On the 13th she vomited, and by the 17th vomiting had become more frequent. The bowels acted on the 16th. The pain was immediately above the umbilicus. On admission to hospital on April 21st there was found a swelling the size of a hen's egg in the right groin. It was dull, fluctuant, and irreducible. An operation was performed. The hernial sac had three locuti containing inflammatory fluid. A piece of bowel was found strangulated for a part of its circumference in the internal ring. There was a well-marked sulcus, but no ulceration. Radical cure was performed, and recovery followed. The case is interesting as being in the inguinal region, this form of hernia generally being femoral. It seems to have been a hernia into the canal of Nück. It only formed an obvious swelling after inflammatory effusion had distended the sac. The symptoms of obstruction were more marked than usual. Russel S. Fowler[6] gives a full account of partial enterocele based upon a study of all reported cases and of two of his own cases, and appends a full bibliography up to January 1st, 1899. It may occur at any of the hernial sites, or be ventral, or in the supernumerary sac of an interstitial hernia. It may be of the jejunum, ileum, or colon. The intestine included may be a congenital diverticulum or pouch, an acquired diverticulum or pouch, or an acute constriction of a normal portion of the circumference of the bowel. By an acquired pouch is meant one due to a change in the muscular coat leading to hernial protrusion of the mucous membrane from the gaseous pressure in the interior of the bowel, or a traction pouch due to a peritoneal adhesion. Treves's paper is, of course, quoted, in which it was shown that in one-third of the cases the symptoms were in no way different from ordinary strangulated hernia; in the other two-thirds of the cases the symptoms were less severe than in ordinary hernia. In some cases the bowels are moved naturally daily, in others with medicine. Anderson's interesting case is alluded to. In that, a strangulated ordinary femoral hernia was operated upon without relief of symptoms. A laparotomy showed a partial enterocele in the left obturated foramen, which ruptured on withdrawal. The bowel was resected. The size of the tumour is always small, unless rupture of the bowel has occurred. The size is also dependent upon the quantity of fluid, if any, present in the sac. Vomiting is not always present, and rarely becomes fæcal. The bad prognosis depends a good deal on the difficulty of diagnosis. In nearly 50 per cent. of the reported cases a diagnosis was not made. All cases not operated upon, and reported, have died. Cases, however, may have been successfully treated by taxis, and their nature, therefore, never discovered. Reduction *en masse* has occurred several times. The only cases that recover are those diagnosed and operated upon early.

[1] Lancet, Aug., 1899. [2] Amer. Jour. of Med. Sci., June, 1899; and Cent. f. Chir., 1898, No. 41. [3] New York Med. Jour., June, 1899. [4] Brit. Med. Jour., June, 1899. [5] Lancet, 1899. [6] Annals of Surgery, May, 1899.

